# Psychological mechanisms and configurational pathways of participation in personal transportation carbon trading: an environment–cognition–intention perspective

**DOI:** 10.3389/fpsyg.2025.1677832

**Published:** 2025-12-19

**Authors:** Xiaohui Wu, Jiani Zhao, Shuzhen Li, Shuchao Cao, Yuji Shi, Meiling He

**Affiliations:** School of Automotive and Traffic Engineering, Jiangsu University, Zhenjiang, China

**Keywords:** personal transportation carbon trading, participation intention, psychological mechanism, configurational pathways, low-carbon travel, environmental sustainability

## Abstract

**Introduction:**

Personal transportation carbon emissions pose a key challenge to environmental sustainability, and establishing a personal transportation carbon trading market can help promote the overall low-carbon transformation of society. However, its effectiveness is still constrained by insufficient public participation and a limited understanding of the psychological mechanisms and configurational pathways underlying participation intention.

**Methods:**

This study is based on Social Cognitive Theory (SCT) and explores the key factors influencing residents’ participation intention, as well as the internal connections between these factors, from an Environment–Cognition–Intention perspective. The psychological response mechanisms of individuals’ cognition and intention under external environmental stimuli were examined using structural equation modeling (SEM), and fuzzy set qualitative comparative analysis (fsQCA) was applied to identify configurational pathways that lead to participation intention.

**Results:**

The results indicate that environmental factors, including policy formulation (PF), social support and social norms (SSN), positively influence participation intention (PI). This influence not only directly affects PI but also operates through a series of interrelated cognitive processes, including self-efficacy (SE), perceived usefulness (PU) and perceived ease of use (PEOU), which in turn enhance PI. However, perceived risk (PR) acts as a negative cognitive factor suppressing PI. Furthermore, configurational analysis also clearly demonstrates multiple causal pathways leading to high PI, highlighting that combinations of strong social support and norms, high PU, and low PR are particularly effective.

**Discussion:**

This study provides deeper insight into the psychological mechanisms underlying low-carbon behavior, highlighting how the market activates motivation through economic incentives and reinforces the influence of social norms and perceived collective responsibility on individual behavior. Configurational pathways further suggest that specific combinations of social and cognitive conditions can foster high PI. It provides theoretical support for the development of targeted behavioral incentive strategies to enhance public participation in personal transportation carbon trading and thereby promote environmental sustainability.

## 1 Introduction

The high proportion of carbon emissions in the global transportation sector seriously affects climate change, environmental quality, and poses a challenge to human health (Fan et al., 2022; Yuan et al., 2025). This further exacerbates global warming, leading to frequent extreme weather events and rising sea levels, exacerbating ecosystem degradation and biodiversity loss. These negative impacts not only damage the natural environment, but also pose a serious threat to human safety, health, and living space, hindering the achievement of environmental sustainability goals and the coordinated development of society and the environment (Du et al., 2022; Liu et al., 2024). With the acceleration of urbanization and the surge in car ownership, individual travel behavior has become an increasingly significant source of emissions (Wang et al., 2024). Thus, it is crucial to understand how to motivate individuals to adopt low-carbon travel behaviors and environmentally responsible intentions.

The behavioral transformation of individuals in transportation modes, especially the transition to low-carbon modes, often relies on strong external incentives or internal motivation to overcome the behavioral inertia brought by existing habitual paths (Steg and Vlek, 2009). Establishing a personal carbon trading market (PCT) with economic incentives can activate such motivation, supporting stable low-carbon intentions and wider pro-environmental behavior, thereby promoting environmental sustainability. The Personal Carbon Market (PCT) was first proposed by scholars Eyre (2010) and Fawcett (2010), and since then, scholars have continuously expanded the application scenarios of PCT from different perspectives such as mechanism design (Nie et al., 2022; Tanner et al., 2024), distribution fairness (Balzer et al., 2023; Fan et al., 2016), and public acceptance (Tan et al., 2019; Uusitalo et al., 2021). However, research still insufficiently addresses the coupling of individual travel behavior heterogeneity and carbon market dynamics, and rarely reveals how environmental changes, personal cognition, and behavior interact. In particular, the psychological mechanisms driving sustained PCT participation remain underexplored. Existing literature rarely starts from the theoretical perspective of the “environment–cognition–intention” tripartite interaction, focusing on individuals’ cognitive evaluation, motivation generation, and behavioral adjustment configurational pathways under specific environmental sustainability stimuli. It is difficult to explain why individuals exhibit different intention to participate and behavioral responses when faced with similar mechanism designs. The design optimization at the level of excessive reliance on mechanisms neglects the psychological foundation and behavioral logic behind public participation, which may weaken the long-term sustainability of PCT and thus affect its actual effectiveness in promoting low-carbon transformation and achieving environmental sustainability goals.

Based on social cognitive theory and from the perspective of environmental psychology, this article constructs an “environment–cognition–intention” ternary interaction model to systematically explore the psychological cognitive changes of individuals in response to external environmental stimuli and their impact mechanism on low-carbon behavioral intentions. This study revolves around three core questions: (1) Through which psychological mechanisms do environment affect individuals’ intention to participate in low-carbon travel? (2) How does an individual’s cognitive evaluation of low-carbon travel change when faced with different environmental stimuli, and how does it regulate their travel decisions? (3) Is there an interactive effect between environment and individual cognition, jointly influencing the formation process of low-carbon behavior? This article emphasizes the psychological changes that affect individuals’ intention to participate, and innovatively reveals how external incentives can be internalized into sustainable behavioral choices through cognitive channels. This study aims to explore the potential driving factors for residents to choose low-carbon travel and actively participate in the personal transportation carbon trading market, providing theoretical support for improving the conversion rate of public low-carbon behavior. At the same time, the transformation of public environmental protection travel modes and the increase in market PI will help achieve the strategic goals of carbon peak and carbon neutrality, providing theoretical basis and suggestions for supporting ecological civilization construction and environmental sustainability.

The remainder of this paper is organized as follows. Section 2 reviews the relevant literature and develops the theoretical hypotheses, with particular emphasis on the psychological processes underlying factor interactions. Section 3 describes the research methodology, including questionnaire design and data collection. Section 4 presents the results, including hypothesis testing, identification of the environmental psychology mechanisms influencing PI, and an exploration of three configurational pathways leading to high PI. Finally, Section 5 provides the discussion and conclusion, summarizes the key findings, and offers recommendations for future research and practical application.

## 2 Literature review and theoretical hypotheses

### 2.1 Personal transportation carbon trading market

The personal transportation carbon trading market introduces personal transportation data into PCT, aiming to guide individuals to prioritize more environmentally friendly modes of transportation through economic incentives and promote the low-carbon transformation of the transportation sector (Xia et al., 2023; Yang et al., 2025). The core of this market system is that individuals can accumulate carbon credits or credits by choosing low-carbon modes of transportation such as public transportation, cycling, or walking. These carbon credits or credits can be traded on the market, bringing economic benefits to individuals (Liu et al., 2025; Liu et al., 2022; Zhao et al., 2024).

### 2.2 Environment

The impact of environment on individual behavior is profound (Feng et al., 2021). In this study, the environment is defined as the sum of external factors that influence individuals to prioritize low-carbon transportation and actively participate in the personal transportation carbon trading market. It mainly consists of two parts: PF and SSN. Research has shown that when faced with new institutional changes, the public often exhibits a certain degree of skepticism and wait-and-see behavior due to the psychological mechanisms of risk avoidance and cognitive inertia (Starkey, 2012). However, with the continuous attention of the government to climate change and environmental issues, the formulation of environmental policies tends to be clear and systematic, gradually releasing a firm signal to promote environmental sustainability. This policy orientation not only increases public attention to environmental issues, but also deepens individuals’ understanding and empathetic awareness of the connection between their own behavior and environmental outcomes, thereby stimulating their intrinsic motivation to adopt a more environmentally friendly lifestyle. When policy content is more operable, fair, and stable in the long run, it helps to reduce the public’s perceived uncertainty and resistance toward new mechanisms (Bartczak et al., 2025; Lindvall et al., 2025). Clear and credible policy objectives can enhance the public’s trust in the government’s environmental commitments, thereby promoting their gradual acceptance and active participation from the initial wait-and-see attitude. In other words, the continuous improvement of environmental policies is not only a progress at the institutional level, but also shapes the public’s recognition of low-carbon lifestyles at the psychological level, promoting their understanding and support for new mechanisms through practical actions, and forming a positive interaction.

Social support and norms refer to the influences received by individuals from the external environment during social interactions, including emotional encouragement, behavioral imitation, informational guidance, and group norm constraints. These factors collectively affect an individual’s attitudes, decisions, and behavioral choices (Al-Shehari et al., 2024; Si et al., 2022). Media platforms strengthen the feasibility and importance of low-carbon consumption in the public’s minds by continuously promoting the concept of green travel, such as new energy vehicles, shared bicycles, carpooling (Cudjoe et al., 2024). In addition, if peers, family members, friends, and opinion leaders in social circles widely advocate or practice low-carbon transportation, it will invisibly create behavioral implications and psychological pressure on individuals (Abdullah et al., 2016; Kavota et al., 2020). For example, when people around them generally choose low-carbon transportation such as subways and buses, individuals tend to imitate and adopt similar modes of transportation due to seeking group identity, reducing social distance, or catering to mainstream trends. This phenomenon of behavioral convergence based on social support and norms not only enhances individuals’ acceptance of low-carbon transportation, but also subtly increases their intention to participate in the personal transportation carbon trading market. Therefore, the following hypothesis is proposed:

*H1*: Policy formulation positively influences participation intention.

*H2*: Social support and norms positively influence participation intention.

### 2.3 Individual cognition

SE refers to individuals gradually constructing expectations for the potential outcomes of specific behaviors in specific situations through continuous interaction based on their observations and practical experiences of different social patterns (Bandura, 1986). In this study, SE specifically refers to residents’ confidence in obtaining economic benefits and promoting low-carbon transportation through participating in the personal transportation carbon trading market, jointly contributing to environmental sustainability (Jiang and Gossage, 2025). When individuals have a high sense of SE, they are more likely to believe that they can overcome obstacles such as inconvenient travel, difficulty in route planning, and increased time costs, and are therefore more willing to choose green modes of transportation such as walking, cycling, or public transportation. On the contrary, if individuals have doubts about their own behavioral abilities, even if they agree with environmental protection concepts, they may choose to rely more on high carbon transportation methods such as private cars due to a lack of confidence in taking action. SE not only affects the onset of behavior, but also relates to the persistence and adaptability of behavior (Li et al., 2024; Phipps et al., 2013). When participating in the personal transportation carbon trading market, individuals who firmly believe that they can control carbon emissions through reasonable travel planning are more likely to participate in the market for a long time, adapt to quota adjustments, and actively seek lower carbon travel methods, thereby internalizing low-carbon behavior into daily habits. In addition, the improvement of SE will enhance individuals’ sense of environmental responsibility and belonging, and stimulate their recognition of environmental sustainability goals. Under the dual influence of policy support and social advocacy, if the public generally establishes the belief that “I can make changes for environmental protection,” green behavior will no longer be the choice of a few people, but will gradually evolve into a common action of mainstream society (Guo et al., 2021; Li et al., 2024; Schunk and DiBenedetto, 2020).

According to the theory of information asymmetry, individuals with different abilities to obtain key information will face varying degrees of PR when participating in new policies, and their grasp of risk will lead to different behavioral tendencies (Bergh et al., 2019). Perceived Risk Theory (PRT) proposed by Cunningham (1967) suggested that PR is composed of the uncertainty of loss occurrence and the severity of potential outcomes. The personal transportation carbon trading market is an innovative low-carbon emission reduction mechanism that is still in its early stages of promotion. Issues such as unstable policy mechanisms and insufficient information transparency will strengthen the public’s sense of uncertainty and unfamiliarity with the market, thereby amplifying concerns about potential economic costs and behavioral failures (Li et al., 2018; Shen and Li, 2023). When the public is unable to assess the consequences of their actions or lacks confidence in institutional rules, self-protection mechanisms will prompt them to choose to observe or avoid possible failures or losses. This psychological reaction not only reduces an individual’s behavioral intention, but may also have a negative impact on the promotion of the entire market mechanism. Therefore, we assume the following:

*H3*: Self-efficacy positively influences participation intention.

*H4*: Perceived risk negatively influences participation intention.

In addition to SE and PR, an individual’s assessment of technological attributes also plays a crucial role in behavioral decision-making. The Technology Acceptance Model (TAM) provides two dimensions for understanding an individual’s adoption of new technologies: PEOU and PU (Chau, 1996; Davis, 1989). In this study, PEOU refers to the subjective judgment of the public on the convenience of participating in the personal transportation carbon trading market process, the ease of operation, and the degree of information acquisition and understanding; PU refers to the subjective evaluation of the public’s adoption of low-carbon lifestyles, particularly through participation in the market, which can effectively reduce transportation carbon emissions, improve surrounding air quality, mitigate climate change, and promote environmental sustainability (Wang et al., 2017). Yuan et al. (2023) pointed out that users’ perceptions of the usefulness and ease of use of a specific technology can predict their attitudes toward that technology, which in turn can affect their intention to adopt and ultimately lead to actual adoption behavior. When the public believes that the personal transportation carbon trading market is easy to operate, has clear processes, and has no barriers to information acquisition, their psychological entry threshold for this mechanism will be significantly reduced. This perception of operational feasibility can effectively alleviate individuals’ common technological anxiety when facing new systems, thereby enhancing their confidence in participating in behavior (Chen et al., 2022; Xu et al., 2018). With the reduction of cognitive burden, individuals are more likely to develop positive attitudes and are willing to try to engage and participate in this institutional arrangement. If individuals can clearly perceive that their participation in low-carbon behavior has a practical positive impact on environmental goals such as improving air quality and mitigating greenhouse effects, a clear psychological connection of “behavior–environment improvement” and empathetic awareness of shared environmental responsibility will be established. This positive cognition of the effectiveness of market mechanisms will enhance their subjective recognition of behavioral values, stimulate their sense of environmental responsibility and goal-oriented awareness. This type of perceived “usefulness” evaluation not only strengthens an individual’s intrinsic motivation, but also enhances their sense of value identification in social norms, making them more inclined to practice environmental behavior in the long term and continuously (Jaiswal et al., 2021). Therefore, the following hypothesis is proposed:

*H5*: Perceived usefulness positively influences participation intention.

*H6*: Perceived ease of use positively influences participation intention.

### 2.4 Impact of the environment on individual cognition

In the context of promoting the personal transportation carbon trading market, individual behavioral intentions are not only directly influenced by external environment such as institutional design and social atmosphere, but also deeply regulated and driven by a series of internal psychological mechanisms (Li et al., 2024; Sun et al., 2025). Specifically, policy-making (such as economic incentives and low-carbon certification systems) and social support and norms (such as increasing public green awareness, social dissemination of low-carbon behavior, and expectations from others) jointly construct the external environment in which individuals operate. When the environment is characterized by fairness, transparency, and guidance, it can effectively enhance individuals’ SE, that is, strengthen their confidence in understanding, mastering, and successfully executing carbon trading behaviors. Once this belief in ability is established, it will greatly stimulate individuals’ motivation to take action, enhance their self-identity toward low-carbon behavior, and encourage their active participation (Pan et al., 2025; Yang et al., 2024). In addition, in the early stages of promoting new mechanisms, individuals often have uncertainty and loss expectations toward unknown systems, which constitutes the psychological basis for their PR. If policies can reduce public concerns about potential economic, time, or privacy costs through technological safeguards (such as blockchain to enhance information transparency and security) and institutional norms (such as data privacy protection), it can significantly alleviate their cognitive anxiety and thus increase their intention to accept (Al-Shehari et al., 2024). Wang et al. (2025) pointed out the key role of data security management in reducing individual participation risks, while Al-Shehari et al. (2024) further pointed out that blockchain technology effectively alleviates individuals’ concerns about participation risks and affects their intention to participate by improving data security and transparency. Meanwhile, risk perception is closely related to a sense of responsibility. Research has found that environmental risk perception plays a reinforcing role between environmental values and behavior, stimulating individuals’ sense of moral responsibility and prompting them to respond to ecological challenges through action (Huo et al., 2025; Liobikienė and Juknys, 2016).

Wang et al. (2022) found through game theory experiments that public awareness of fairness in policy implementation significantly affects their intention to participate in green behavior decision-making. Specifically, when policy systems have fair procedural design, clear implementation processes, and reasonable technical support, the public is more likely to trust and affirm the system. This external institutional environment enhances individuals’ subjective perception of the “usefulness” of policies, that is, they believe that policies can effectively achieve environmental improvement goals, thereby psychologically stimulating their recognition of the value of green systems and enhancing their motivation and commitment to participate in the personal transportation carbon trading market (Riemer et al., 2025). This indicates that institutional fairness and process transparency can promote the implementation of public environmental behavior through the psychological pathway of PU, and provide support for building a fair and sustainable green governance mechanism (Chen C. et al., 2025). Xu et al. (2018) pointed out that PEOU, as a “cognitive bridge” between external system design and individual action, is mainly reflected in reducing the cognitive burden and operational anxiety of public participation when studying the acceptance of autonomous vehicle. In the personal transportation carbon trading market, an understandable and operable institutional structure can reduce public decision-making hesitation, making individuals no longer feel isolated and pressured when facing complex social mechanisms, but rather more likely to view them as approachable and collaborative social systems. This psychological comfort promotes individuals to actively participate in behavioral decision-making, thereby enhancing the stability and sustainability of their behavior (Zhu et al., 2025). Based on these findings, the following hypotheses are proposed:

*H7*: The environment influences participation intention through individual cognition.

**FIGURE 1:**
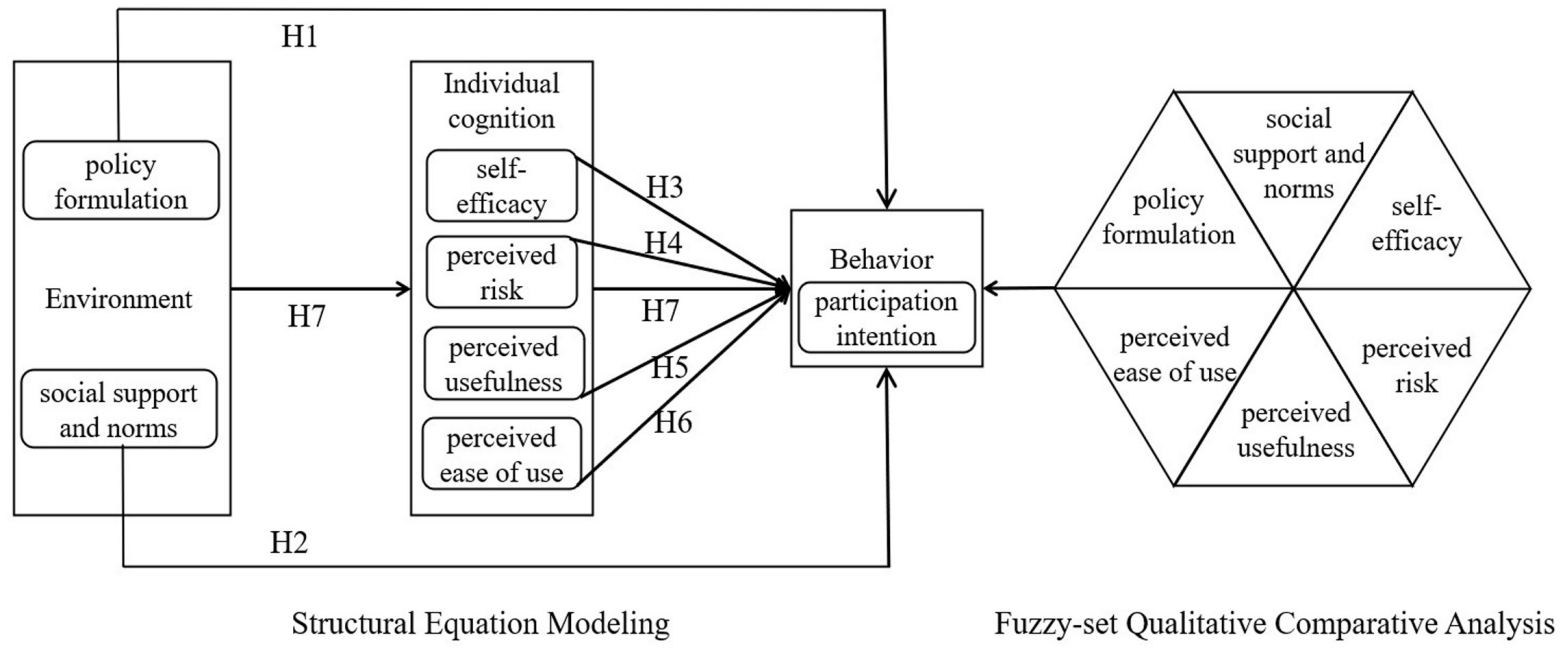
Research model of the personal transportation carbon trading market participation intention.

The analytical framework of this study is modified and depicted as in Figure 1.

## 3 Materials and methods

### 3.1 Methods

This study draws on the environment cognition intention perspective of SCT, supplemented by the TAM and PRT, to construct a comprehensive theoretical framework for analyzing the psychological mechanisms of residents’ participation in personal transportation carbon trading under policy stimulus and institutional innovation scenarios. The core advantage of SCT lies in its ability to simultaneously handle dynamic interaction mechanisms between multiple factors such as external policy environment, social norms, self-efficacy, and experience feedback. It is suitable for individual transportation carbon trading mechanisms that are still in the cognitive cultivation stage and highly dependent on policy guidance (Akter, 2023; Schmitt et al., 2022). In contrast, Theory of Planned Behavior (TPB) mainly explains behavioral intention based on a relatively stable attitude norm perception control structure, while Norm Activation Model (NAM) emphasizes the activation path of moral responsibility. Both theories have sufficient explanatory power in exploring individual behavior in many conventional contexts. However, for the context in which policy pilots, institutional embeddings, and technological applications jointly play a role that this study focuses on, the theoretical assumptions have relatively limited coverage of the dynamics, situational dependence, and learning feedback mechanisms of the behavior formation process. Therefore, it is difficult to fully reflect the cognitive adjustment process of individuals under multiple external stimuli.

### 3.2 Questionnaire design

Since the personal transportation carbon trading market has not yet been implemented, the study collects data on residents’ participation intentions, allowing analysis of the underlying cognitive and environmental mechanisms that may shape future behavior. The questionnaire was designed with the main purpose of measuring residents’ understanding of and attitudes toward the personal transportation carbon trading market, as well as identifying the corresponding influencing factors. The questionnaire consisted of three main parts: general information of residents, residents’ intention to participate, and the influencing factors of PI, including the environment and personal factors, as shown in Figure 1. In this study, residents’ intention to participate served as the dependent variable, while the influencing factors served as independent variables. A 5-point Likert scale was provided for the dependent and independent variables, where “1” represented “strongly disagree,” “3” represented the midpoint scale and a “neutral” attitude toward these statements, and “5” represented “strongly agree.” This survey questionnaire mainly drew on the survey conducted by Sun et al. (2025) on residents’ participation in digital green public services, as well as the survey conducted by Schunk and DiBenedetto (2020) using SCT to explain the process of motivational behavior. Please refer to Table 1 for detailed scale questions.

**TABLE 1:** Explanation and statistics of survey questionnaire variables.Latent variable

	Measurement question	References
Policy formulation (PF)	PF1 The personal transportation carbon trading market is relatively fair.	(Chen Z. et al., 2025; Mzembe and Filimonau, 2024)
	PF2 The personal transportation carbon trading market can effectively reduce carbon emissions.	
	PF3 The rules and objectives of the personal transportation carbon trading market are clear and explicit.	
Social support and norms (SSN)	SSN1 The monitoring mechanism of the personal transportation carbon trading market is scientifically reasonable.	(Hwang and Venter, 2025; Si et al., 2022)
	SSN2 Having corresponding sound laws and regulations in the personal transportation carbon trading market can increase the intention to participate.	
	SSN3 The personal transportation carbon trading market can increase participation intention through authoritative publicity methods.	
Self-efficacy (SE)	SE1 I have the ability to reduce my carbon emissions and gain economic benefits by changing my daily behavior.	(Sun et al., 2025; Du et al., 2021)
	SE2 I am able to understand and use the rules and tools of the personal transportation carbon trading market correctly.	
	SE3 I can persist in participating in the personal transportation carbon trading market for a long time and integrate it into my daily life.	
Perceived risk (PR)	PR1 If an individual chooses a high carbon mode of transportation, they will need to incur additional expenses to purchase carbon emission quotas that exceed them.	(Nguyen-Phuoc et al., 2024; Li et al., 2018)
	PR2 There may be risks of complex operations or high time costs.	
	PR3 There may be insufficient liquidity of carbon quotas to purchase or sell them in a timely manner when needed.	
Perceived usefulness (PU)	PU1 The personal transportation carbon trading market effectively reduces carbon emissions and provides support for environmental behavior.	(Cudjoe et al., 2024; Kavota et al., 2020)
	PU2 Participating in the personal transportation carbon trading market can generate actual economic benefits and is valuable.	
	PU3 The personal transportation carbon trading market has a positive impact on environmental protection and is meaningful.	
Perceived ease of use (PEOU)	PEOU1 The rules and operational procedures of the personal transportation carbon trading market are simple and easy to understand.	(Cudjoe et al., 2024; Kavota et al., 2020)
	PEOU2 The tools and platforms required to participate in the personal transportation carbon trading market are convenient to use.	
	PEOU3 The participation threshold of the personal transportation carbon trading market is relatively low and suitable for the general public to participate.	
Participation intention (PI)	PI1 Willing to actively participate in the personal transportation carbon trading market and integrate it into my daily life.	(Li et al., 2016; Wang et al., 2022)
	PI2 Willing to proactively learn about relevant information in the market and adjust my behavior according to policy requirements.	
	PI3 Willing to recommend a personal transportation carbon trading market to family and friends and encourage them to participate together.	

### 3.3 Data collection and analysis

The data were collected through an online questionnaire distributed via Wenjuanxing and shared across multiple WeChat groups, enabling participation from respondents in different regions of China. This approach helped obtain a diverse sample of residents with varying travel behaviors and intentions in the context of low-carbon transportation.

An electronic poster containing an explanatory letter, outlining the purpose of the survey and the distributing institution, was provided to all potential participants. The questionnaire was collected anonymously, and participants were encouraged to share the survey link with other residents in their social networks, helping broaden the respondent base and improve sample diversity. A total of 873 responses were received, among which 784 were valid, resulting in a validity rate of 89.81%. Table 2 presents some preliminary statistical data.

**TABLE 2:** Basic information of valid samples in the survey questionnaire.

Variable	Category	Number	Proportion
Gender	Male	379	48.36
	Female	405	51.64
Age	14–20	22	2.9
	21–30	297	38.01
	31–40	274	35.00
	41–50	111	14.28
	51–60	53	6.80
	≥ 61	27	3.01
Educational background	Primary school diploma and below	41	5.23
	Middle school diploma	118	15.05
	High school diploma	229	29.21
	university diploma	313	39.92
	Graduate school diploma	83	10.59
Income(CNY)	<2,000	31	3.95
	2,000–6,000	87	11.10
	6,000–10,000	261	33.29
	10,000–14,000	202	25.77
	14,000–18,000	120	15.31
	>18,000	83	10.58

In order to reduce sample bias in data collection, the analysis controlled for four observable variables, gender, age, education, and income, through propensity score inverse probability weighting (IPW). The above variables have been widely confirmed in existing literature as core demographic representations of environmental awareness and pro environmental tendencies (Chen Z. et al., 2025; Cudjoe et al., 2024). These variables are not only highly correlated with the willingness to participate in the survey, but also systematically reflect the observable performance of environmental awareness in reality. Therefore, by incorporating them into the IPW model, we have effectively controlled for potential selective biases caused by environmental awareness and other factors at the observable level. Although residual confounding of unobserved dimensions cannot be completely ruled out, the selected variables have covered their main pathways of action, providing a theoretically reasonable and empirically robust adjustment. The IPW test results in Table 3 reflect that when using IPW to handle voluntary sample bias, the weighted balance of each treatment variable has been significantly improved. The standardized mean differences of the four variables are controlled within 0.1. Combined with the mean and standard deviation results obtained from Bootstrap repeated sampling, it can be seen that the balance indicators of different variables fluctuate less after repeated perturbations, indicating that the weighted sample distribution has high robustness.

**TABLE 3:** Propensity score inverse probability weighting (IPW) reduces data bias.

Variable	Covariate	5-fold cross-validation	PS Range	IPW Range	SMD	SMD ± SD
Gender	Age; educational background; income	0.483 ± 0.027	0.266 –0.775	0.720–1.690	0	0.000 ± 0.000
Age	Gender; educational background; income	0.330 ± 0.028	0.210–0.636	0.790–1.730	−0.035	−0.0,345 ± 0.005
Educational background	Age; gender; income	0.296 ± 0.014	0.000–0.917	0.070–11.210	−0.085	−0.097 ± 0.036
Income	Age; gender; educational background;	0.146 ± 0.033	0.002– 0.251	0.480–1.470	−0.005	−0.007 ± 0.004

## 4 Results

### 4.1 Validity, reliability and goodness-of-fit tests

SPSS 27.0 was used to test the reliability and validity of the questionnaire data. Firstly, Cronbach’s alpha was used as the reliability indicator. The coefficients of the seven latent variables were 0.886, 0.917, 0.919, 0.902, 0.911, 0.928, and 0.917, respectively, all exceeding the 0.6 threshold, indicating good internal consistency. Next, the KMO test (0.915) and Bartlett’s test of sphericity (*p* < 0.001) confirmed the suitability of the 21 items for factor analysis. Finally, principal component analysis extracted 7 common factors, explaining 85.145% of the total variance (see Table 4), indicating strong construct validity.

**TABLE 4:** Results of exploratory factor analysis (EFA) and confirmatory factor analysis (CFA).

EFA					CFA		
Latent variable	Question number	Factor loading	Eigenvalue	Cumulative variance explained(%)	Factor loading	AVE	CR
PF	PF1	0.821	10.357	49.308	0.815	0.733	0.895
	PF2	0.750			0.824		
	PF3	0.746			0.928		
SSN	SSN1	0.843	1.612	56.976	0.866	0.784	0.917
	SSN2	0.820			0.863		
	SSN3	0.810			0.928		
SE	SE1	0.870	1.429	63.811	0.896	0.807	0.926
	SE2	0.829			0.863		
	SE3	0.817			0.930		
PR	PR1	−0.868	1.251	69.757	0.838	0.747	0.898
	PR2	−0.840			0.849		
	PR3	−0.817			0.901		
PU	PU1	0.831	1.193	75.463	0.873	0.748	0.900
	PU2	0.825			0.867		
	PU3	0.799			0.845		
PEOU	PEOU1	0.857	1.021	80.296	0.894	0.817	0.931
	PEOU2	0.811			0.863		
	PEOU3	0.808			0.953		
PI	PI1	0.823	1.020	85.145	0.855	0.789	0.918
	PI2	0.801			0.839		
	PI3	0.730			0.970		

Confirmatory factor analysis was performed using AMOS 29.0, as shown in Table 5. Model fit indices showed good fit: CMIN/DF = 2.619 < 3, RMSEA = 0.052 < 0.08, and IFI, TLI, and CFI all exceeded 0.9. The average variance extracted (AVE) for each latent variable was above 0.5, and composite reliability (CR) exceeded 0.7. All latent variables were significantly correlated (*p* < 0.001), and the square roots of AVE were greater than the corresponding inter-factor Pearson correlations, indicating good discriminant validity.

**TABLE 5:** Results of validity test for differences in various factors.

Variables	PF	SSN	SE	PR	PU	PEOU	PI
PF	**0.858**	**0.884**	**0.895**	**0.863**	**0.868**	**0.902**	**0.891**
SSN	0.583***						
SE	0.587***	0.470***					
PR	−0.548***	−0.504***	−0.445***				
PU	0.588***	0.508***	0.557***	−0.475***			
PEOU	0.561***	0.580***	0.522***	−0.496***	0.536***		
PI	0.620***	0.574***	0.563***	−0.567***	0.567***	0.560***	

***Indicates significance at the 1% level. The diagonal values represent the square root of the AVE for each factor.

### 4.2 Path analysis and hypothesis testing

The path relationships in the theoretical model are shown in Table 6. Assuming that both H1 and H6 were supported (*p* < 0.05), PR had a significant negative impact on PI (β = −0.253, *p* < 0.001), with the highest absolute standardized path coefficient, indicating that it was the primary factor inhibiting individual PI. When residents felt that participating in the mechanism might bring uncertainty, privacy breaches, resource waste, or other potential risks, they were more likely to hold a reserved attitude toward the mechanism and reduce their motivation to participate. The other five factors had a significant positive impact on PI, including PF (β = 0.220, *p* < 0.001), SSN (β = 0.217, *p* < 0.001), SE (β = 0.225, *p* < 0.001), PU (β = 0.181, *p* < 0.001), and PEOU (β = 0.147, *p* < 0.001). These results demonstrated that the cognitive value of the system, operational confidence, and external support were key factors in enhancing residents’ intention to participate in the personal transportation carbon trading market.

**TABLE 6:** Results of path relationship test for SEM.

Path relationship	β	S.E.	C.R.	*p*
PI < — PF	0.220	0.039	5.508	***
PI < — SSN	0.217	0.035	5.417	***
PI < — SE	0.225	0.038	5.576	***
PI < — PR	−0.253	0.037	−6.410	***
PI < — PU	0.181	0.034	4.519	***
PI < — PEOU	0.147	0.032	3.780	***

***Indicates significance at the 1% level for the SEM path coefficients.

### 4.3 Intermediary effect test

**FIGURE 2:**
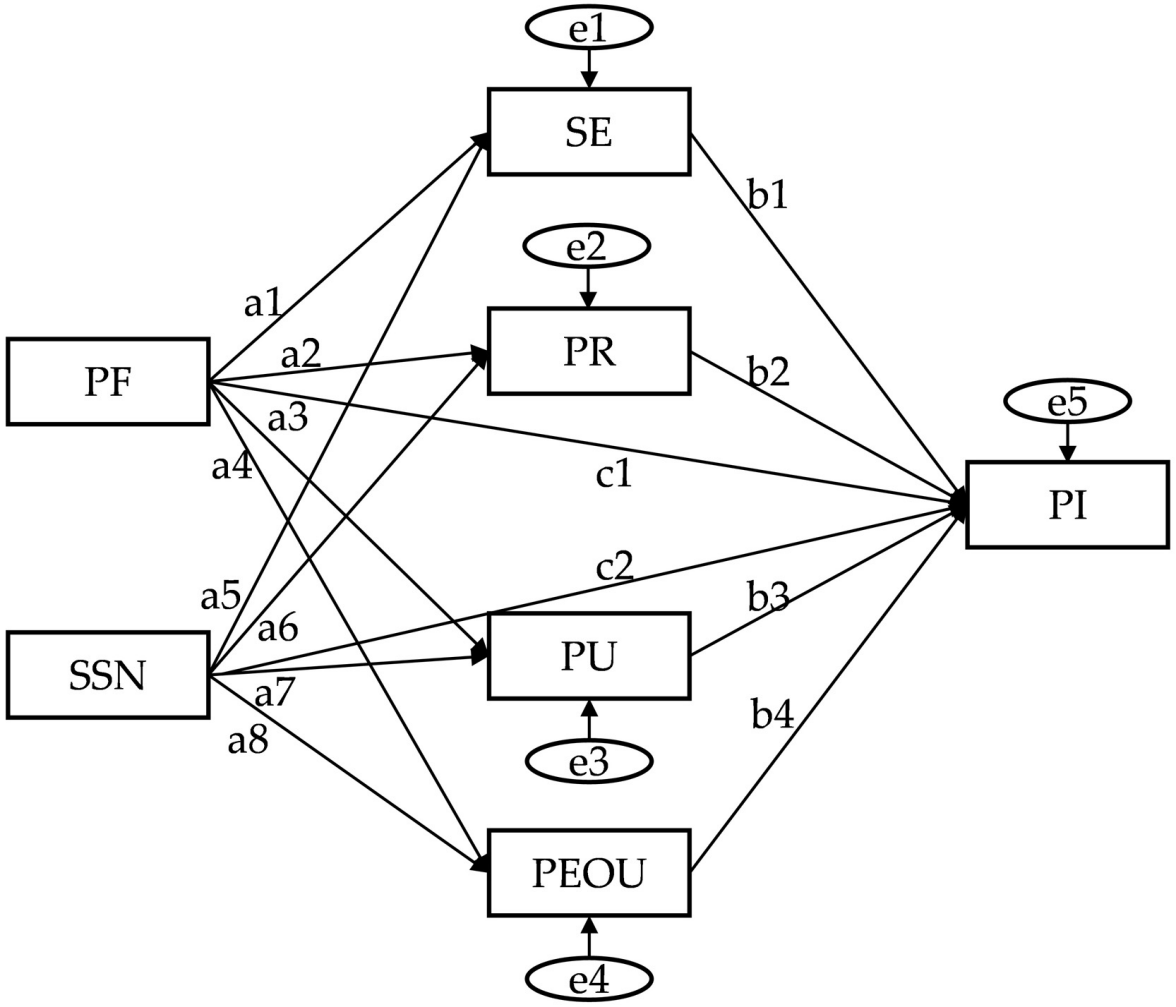
Path diagram of mediating variable influence.

The mediation effect was tested using AMOS 29.0 and Bootstrap method (5,000 samples; 95% bias corrected confidence interval). As shown in Figure 2, personal cognition was seen as a mediating variable between the environment and PI. Table 7 showed that the 95% confidence intervals of eight intermediary paths did not include zero, and all intermediary paths reached a significant level (*p* < 0.01). Therefore, the H7 was supported, indicating that individual cognition mediates the relationship between the environment and intention to participate. Among these, Path ③ exhibited the strongest mediation effect, with the highest absolute value and the largest relative contribution. This path indicated that when residents believed that policy design was reasonable, implementation was transparent and reliable, they were more likely to believe that their rights and interests in the participation process would not be violated, and the process was controllable and predictable, thereby significantly reducing concerns about the ambiguity, complexity, and uncertainty of the mechanism. This was reflected as an institutional trust mechanism (Bachmann and Inkpen, 2011), where good policy support enhanced residents’ trust in the system, thereby alleviating concerns about potential losses such as privacy breaches and difficulties in safeguarding their rights, and increasing their intention to cooperate with the new system.

**TABLE 7:** Direct mediation test.

Path relationship	Estimate	Lower	Upper	*p*
① PF→SE→PI	0.074	0.029	0.117	0.002
② SSN→SE→PI	0.023	0.011	0.053	0.001
③ PF→PR→PI	0.074	0.043	0.112	0.002
④ SSN→PR→PI	0.049	0.030	0.081	0.001
⑤ PF→PU→PI	0.059	0.025	0.109	0.001
⑥ SSN→PU→PI	0.036	0.023	0.068	0.002
⑦ PF→PEOU→PI	0.046	0.018	0.085	0.003
⑧ SSN→PEOU→PI	0.047	0.015	0.090	0.002
**Path proportion**				
① Proportion	0.094	0.045	0.160	0.002
② Proportion	0.047	0.014	0.071	0.001
③ Proportion	0.100	0.056	0.163	0.001
④ Proportion	0.067	0.032	0.103	0.002
⑤ Proportion	0.076	0.032	0.141	0.001
⑥ Proportion	0.050	0.015	0.087	0.002
⑦ Proportion	0.059	0.020	0.112	0.003
⑧ Proportion	0.061	0.018	0.116	0.002

Based on the above results, it can be observed that SE plays a key mediating role in multiple pathways of action, and its influence runs through the transmission process of the independent variable to the dependent variable. To further clarify the role of SE in different psychological mechanism chains and its relative position in the pathway, this study used chain mediation analysis to examine how independent variables PF and SSN affect SE through multiple cognitive variables such as PR, PEOU, and PU, and ultimately conducted a detailed test on the indirect effects of SI. Furthermore, we add effect decomposition and report the indirect effects of each path. By breaking down the overall mediation effect into multiple chain paths with clear psychological logic order, it is possible to more specifically identify the functional positioning and priority of different cognitive variables in the mechanism of action, thereby enhancing the explanatory power and theoretical contribution of the model. According to Table 8, the indirect effects of the six chain paths are significant. Specifically, the indirect effect of path a. is 0.030, with SE having a negative impact on PR (−0.189); The indirect effect of path c. is 0.036, and PEOU positively predicts SE (0.271); The indirect effect of path e. is 0.007, and the path coefficients of each link are positive. For SSN, the indirect effect of path b. is the largest (0.043), and SE has a positive impact on PU (0.399); The indirect effect of path d.is 0.034, and PR has a negative effect on PU (−0.270); The indirect effect of path f. is 0.013, and both PEOU → SE (0.352) and SE → PU (0.325) are positive. Overall, both PF and SSN exhibit significant and robust chain mediation effects on SI through multiple paths containing different mediation sequences.

**TABLE 8:** Chain mediation test.

Serial mediation path	Total effect	*c*′	Indirect effect	CI	*p*	Estimate
a. PF → SE → PR → SI	0.638	0.343	0.030	(0.014, 0.051)	0.001	PF → SE: 0.568 SE → PR: −0.189
b. SSN → SE → PU → SI	0.547	0.313	0.043	(0.024, 0.065)	0.001	SSN → SE: 0.420 SE → PU: 0.399
c. PF → PEOU → SE → SI	0.638	0.359	0.036	(0.019, 0.057)	0.002	PF → PEOU: 0.582 PEOU → SE: 0.2,714
d. SSN → PR → PU → SI	0.547	0.283	0.034	(0.019, 0.053)	0.001	SSN → PR: −0.455 PR → PU: −0.270
e. PF → SE → PEOU → PU → SI	0.638	0.303	0.007	(0.003, 0.014)	0.002	PF → SE: 0.568 SE → PEOU: 0.304 PEOU → PU: 0.219
f. SSN → PEOU → SE → PU → SI	0.547	0.243	0.013	(0.006, 0.022)	0.001	SSN → PEOU: 0.552 PEOU → SE: 0.352 SE → PU: 0.325

### 4.4 fsQCA

To overcome the limitations of traditional research limited to single factor analysis and enhance the scientific and explanatory power of research results, this study used fsQCA to systematically explore the multiple influencing factors and their collaborative mechanisms of public PI from a configurational perspective, in order to more comprehensively reveal the essential laws of this complex behavior.

#### 4.4.1 Selection and calibration of variables

Select PF, SSN, SE, PR, PU, and PEOU as antecedent variables, and intention to participate as outcome variable. FsQCA converted six antecedent variables and one outcome variable into a fuzzy set through direct calibration. Specifically, three anchor points were set during the calibration process: 0.95, 0.5, and 0.05, corresponding to the fully dependent, intermediate dependent, and fully nondependent points of the sample data, respectively (Ragin, 2008).

#### 4.4.2 Analysis of necessity conditions

Before conducting a combination analysis of truth tables, the main task was to verify the necessity of individual conditions. According to research standards, the consistency level of a single condition must be above 0.85 to be considered a necessary condition (Ragin, 2008). However, as shown in Table 9, the consistency of each conditional variable did not reach this threshold, indicating that these factors alone cannot motivate individuals to take action to participate in the personal transportation carbon trading market.

**TABLE 9:** Necessity test of various conditional variables in fsQCA.

Conditional variables	Outcome variable	
	PI	∼PI
PF	0.779	0.576
∼PF	0.483	0.731
SSN	0.768	0.594
∼SSN	0.499	0.727
SE	0.798	0.631
∼SE	0.480	0.698
PR	0.256	0.417
∼PR	0.839	0.841
PU	0.785	0.625
∼PU	0.504	0.741
PEOU	0.751	0.485
∼PEOU	0.412	0.730

#### 4.4.3 Configuration pathways analysis

When conducting the configuration pathways analysis, following Ragin (2008)’s suggestion, a consistency threshold of 0.8 was set and the case frequency was set to 3. The final configuration results for the preconditions of the public’s intention to act in the personal transportation carbon trading market were presented in Table 10. The overall consistency was 0.850, and the total coverage rate was 0.755. The consistency of each antecedent condition configuration exceeded 0.6, indicating that the model had a good explanatory power. According to the analysis results of fsQCA, the public’s intention to participate in the personal transportation carbon trading market could be achieved through three configuration pathways, namely C1, C2, and C3.

**TABLE 10:** Preconditions configuration of participation intention.

Configuration	C1	C2	C3
PF	●	●	●
SSN	●		●
SE	⊗	⊗	
PR			⊗
PU		●	●
PEOU			
Original coverage rate	0.620	0.625	0.631
Unique coverage rate	0.070	0.021	0.069
Consistency	0.878	0.894	0.887
Overall coverage rate	0.755		
Overall consistency	0.850		

● Indicates the occurrence of core conditions; ⊗ Indicates that the core condition does not exist; Indicates the occurrence of edge condition; Blank spaces indicate insignificance (they can appear or not).

C1 indicated that high PF and high SSN can induce high PI (consistency = 0.878). The public believed that PF was clear and reasonable, social support was sufficient, and there was a lack of major risk concerns, even if their perception of technological convenience was not strong, it was enough to stimulate their willingness to participate, reflecting the impact of institutional trust and effectively reducing public psychological resistance and concerns. C2 indicated that high SSN, as well as high PU, can generate high PI (consistency = 0.894). Under this configuration pathway, the public chose to trust the system and further realized the practical value of the mechanism (such as economic savings and low-carbon incentives) through the SSN obtained, which enhanced their intention to participate. Even if there were concerns about operational convenience, it did not constitute a substantial obstacle. C3 showed that high PF, high SE, and high PU induced high PI (consistency = 0.887). This indicated that even if social support was not significant, as long as the policy design was good and the public believed that the mechanism was profitable and the risks were controllable, it can still stimulate a strong intention to participate. This configuration pathway emphasized the importance of individuals’ assessment of their own interests and risk judgment. Overall, these three configuration pathways indicated that the quality of PF and the level of risk perception were key factors affecting public PI, while social support and norms and PU played a reinforcing role in different paths, reflecting the psychological basis of different rationality and situational dependence in public participation mechanism decision-making.

## 5 Discussion and conclusion

### 5.1 Discussion

The survey results showed that 71.84% of participants expressed a clear intention to participate, indicating a high level of public recognition of the personal transportation carbon trading market. This not only reflected the intention of residents to actively practice the low-carbon travel concept, but also provided strong data support for promoting carbon market pilot projects and supporting sustainable transportation development.

According to the empirical analysis results in Tables 5, 6, 10, residents’ intention to participate in the personal transportation carbon trading market was significantly negatively affected by PR. In other words, the stronger residents perception of risk, the lower their intention to participate, which was consistent with the initial hypothesis of this article. Moderate risk perception helped individuals rationally assess potential losses and make more cautious decisions. However, if individuals were too sensitive to emerging markets, it might lead to a significant decrease in their intention to participate, thereby missing out on potential opportunities to achieve economic benefits through energy conservation and emission reduction. This result was similar to the findings of Nguyen-Phuoc et al. (2024) and Sun et al. (2025), confirming that PR had a significant negative impact on intention to participate.

The analysis results also indicate that PF, SSN, SE, and PU all have a significant positive impact on residents’ intention to participate. The positive impact of policy-making on PI reflects individuals’ recognition of the legitimacy of the institutional environment and their need for procedural justice. Policies are not only an external incentive mechanism, but also a psychological signal that conveys the government’s emphasis and supports for low-carbon travel, activating the public’s cooperative motivation and conformity tendency. The positive effect of social support and norms is to stimulate individuals’ sense of social belonging and value recognition. When relatives, friends, groups, or society as a whole express support or expectations for low-carbon behavior, individuals tend to align with mainstream attitudes to maintain social relationships or gain recognition. The presence of social norms not only reduces psychological resistance but, through mechanisms such as social influence and behavioral imitation, further encourages residents to adopt low-carbon behavior. Carbon participation regarded as an expression of social responsibility or identity recognition; The role of SE is reflected in stimulating the public’s proactive control psychology, making participation no longer seen as a burden or challenge, but as a behavioral goal that can be led and actively achieved by oneself, providing lasting enthusiasm for subsequent behavior; PU prompts individuals to establish a direct connection between their participation behavior and actual benefits. This includes not only economic incentives such as carbon credits and green subsidies, but also individual expectations for achieving higher-level values such as environmental protection and public interest. In other words, residents are willing to participate because they believe it is meaningful and can bring tangible improvements or long-term benefits for society. PU activates goal-oriented motivation, allowing individuals to continuously gain positive feedback on input and output during the participation process. These are consistent with the research findings of Cudjoe et al. (2024), Hwang and Venter (2025), and Lindvall et al. (2025). The PEOU has the least impact on residents’ intention to participate, which is in stark contrast to the findings of Kavota et al. (2020). This difference may be due to differences in research background. Kavota et al. (2020) primarily focused on real-time reporting of disaster situations through social media, with time being a key factor in enabling the public to take swift action and conduct safety assessments. This study found that although PEOU has a significant impact on residents’ willingness to participate, its impact intensity is relatively small. This phenomenon can be explained by the operational background of PCT, which is different from typical technology adoption scenarios. The promotion of PCT is driven by three factors: policy incentives, economic benefits, and social norms. In this context, residents are more inclined to consider the substantial benefits brought by carbon quotas such as PU and direct economic incentives as key decision variables, slightly reducing the importance of operational convenience—a pattern also observed in Tamilmani et al. (2021) and Luo et al. (2024). In addition, SEM and fsQCA analysis further confirmed that although the path coefficient of PEOU is small, there is still a positive correlation between PEOU and participation intention, but the impact on PI is relatively weak.

In addition, according to the configuration structure analysis results in Table 10, C2 with SSN and PU as the core conditions showed the highest acceptance among residents. This indicates that under the dual effects of positive guidance from the social environment and clear individual perception of the benefits of low-carbon behavior, residents were more willing to participate in the personal transportation carbon trading market. In this configuration pathway, the public not only focuses on whether the market can bring actual economic benefits, but also on whether low-carbon lifestyles can truly improve the air quality around them and promote the overall transformation of environmental sustainability. The visibility and transparency of market operations can effectively alleviate residents’ doubts about the complexity of trading rules and the uncertainty of carbon credit value, thereby improving the information environment and enhancing trading efficiency (Yuan et al., 2025). Therefore, extensive promotion and positive guidance through social media and other platforms cannot only enhance individuals’ understanding and trust in carbon trading mechanisms, but also help stimulate their sense of ecological responsibility and strengthen their psychological identification with the value of low-carbon behavior. This positive interaction between society and individuals will further promote the internalization of low-carbon behavior among residents into stable habits, providing strong support for carbon reduction and environmental sustainability goals in the transportation sector. This conclusion is also consistent with the research results of Si et al. (2022) and Shen and Li (2023).

The configuration pathway C3 focused on PF, SE, and PU, was second only to the C2 in terms of resident acceptance. In this pathway, individual decision-making was mainly based on their own abilities, supplemented by understanding and evaluating policies. When residents believe that they have the ability to control the costs and benefits of low-carbon behavior, and believe that market mechanisms can help achieve their low-carbon living goals, their intention to participate will significantly increase. The policy environment provides external norms and action frameworks, while individuals continuously adjust their behavioral expectations and participation paths through SE and positive perception of market mechanisms. Compared with C1, individuals in the C3 exhibited higher autonomy and exploratory abilities. They were not only willing to have a deep understanding and mastery of carbon trading rules, but also paid attention to their role positioning in institutional practice, especially whether they have the cognitive and operational ability to cope with market complexity. This psychological adaptation pathway based on individual capacity building reflects how people achieve a dynamic balance between external policy guidance and internal cognitive regulation when facing new institutional arrangements. This finding is highly consistent with the research findings of Du et al. (2021), emphasizing the importance of the interaction between self-awareness and the institutional environment in promoting sustainable behavior transformation.

### 5.2 Limitations and future research

Nevertheless, there are still some limitations that should be noted. As the personal carbon trading market for transportation is still in a theoretical stage and lacks practical case data, it was not feasible to conduct a large-scale data analysis. Instead, we adopted a voluntary questionnaire design, focusing on the PI of residents. Consequently, the findings reflect subjective intentions under hypothetical scenarios, and differences may exist between willingness and actual behavior.

To address these limitations, future research could expand the sample scope, adopt longitudinal or experimental designs, and explore pilot programs. For instance, simulated carbon trading environments could be constructed to examine decision-making under quota allocation and price dynamics. Once pilot programs are implemented, tracking the same participants over time could help verify how willingness translates into actual participation.

Moreover, although this study is based on a conceptual framework in China, the findings offer insights into how incentives, social norms, and cognitive factors shape participation, which may be relevant for other regions or countries. Adaptation to local conditions would be necessary, but the principles could inform broader carbon reduction efforts.

### 5.3 Conclusion

This article combines SEM and fsQCA analysis methods to study the influencing factors of public participation in individual transportation carbon trading market intention, further exploring the psychological mechanisms between various factors and various implementation paths to improve PI. Unlike previous studies that focused on reducing greenhouse gas emissions from the production end, this study integrates the concept of carbon trading into the transportation sector from the consumption end and conducts more targeted research on public participation and its driving mechanisms. The research results indicate that a higher level of risk perception significantly suppresses the public’s PI. In contrast, fair and effective policy-making, support from social media, recognition of one’s own abilities, and rational evaluation of market value have all had a positive impact on decision-making.

Based on the above research, the following suggestions are provided for future pilot projects of personal transportation carbon trading markets: guidance strategies should be based on in-depth insights into the public’s choices of transportation modes, sensitivity to carbon costs, and low-carbon incentive responses. Through scientific investigation and research, accurately capturing the dynamic changes in individuals’ cognition, attitudes, and behavioral choices can help design market mechanisms more targeted. In the early stages of market establishment, simplifying the trading process and building a highly transparent and easy to understand trading platform can help alleviate public anxiety and distrust caused by institutional complexity, lower entry barriers, and gradually promote the public to establish a preliminary understanding and interest in the market. In the stage of market promotion, real case sharing, data visualization, and interactive communication tools can help the public more intuitively understand the operating logic of the market and its positive effects on individuals and the environment, thus completing the transition from “bystanders” to “participants” psychologically. At this stage, individuals gradually internalize low-carbon travel as a meaningful social behavior under the influence of social norms and group behavior. In order to further enhance market vitality and institutional fairness, diversified behavioral incentives and support mechanisms should be introduced, such as environmental subsidies, carbon point redemption, tax reductions, etc., to reduce the cost of public participation and enhance the visibility of their behavioral returns. At the same time, market design also needs to fully consider the differences in behavioral ability, information acquisition, and institutional adaptation among different groups of people, especially for those with low income or limited mobility. Differentiated assistance and flexible trading choices should be provided to promote market inclusiveness and accessibility. This not only concerns the operational efficiency of the market, but also reflects the fundamental pursuit of fairness, justice, and environmental sustainability.

From a long-term perspective, with the advancement of technology and the evolution of social cognition, digital tools such as intelligent transportation systems and carbon footprint tracking platforms can further enhance the convenience, participation, and transparency of carbon trading mechanisms, gradually transforming low-carbon travel from short-term policy guidance to a conscious and sustainable lifestyle choice for the public. Through these phased, differentiated, and forward-looking guidance strategies, it is possible to effectively promote positive interaction between individuals and the environment, build a fair, efficient, and widely accepted personal transportation carbon trading market, and guide the public to complete behavioral changes in cognitive transformation, injecting lasting momentum into low-carbon transportation and ecological civilization construction.

## Data Availability

The original contributions presented in this study are included in this article/supplementary material, further inquiries can be directed to the corresponding author.
